# Mapping Bromodomains in breast cancer and association with clinical outcome

**DOI:** 10.1038/s41598-019-41934-3

**Published:** 2019-04-05

**Authors:** Javier Pérez-Pena, Raquel Páez, Cristina Nieto-Jiménez, Verónica Corrales Sánchez, Eva M. Galan-Moya, Atanasio Pandiella, Balázs Győrffy, Alberto Ocana

**Affiliations:** 1 Centro Regional de Investigaciones Biomedicas, Castilla-La Mancha University (CRIB-UCLM), Calle Almansa, 14, 02008 Albacete, Spain; 2 Instituto de Biología Molecular y Celular del Cáncer and CIBERONC. CSIC-Universidad de Salamanca, Campus Miguel de Unamuno, 37007, Salamanca, Spain; 3 Semmelweis University 2nd Department of Pediatrics, 1094 Tűzoltó u. 7-9, Budapest, Hungary; 4 MTA TTK Lendület Cancer Biomarker Research Group, Institute of Enzymology, Magyar tudósok körútja 2, Budapest, Hungary; 5 Experimental Therapeutics Unit, Medical Oncology Department, Hospital Clínico San Carlos, IdISSC, calle profesor Martin Lagos, s/n, 28040 Madrid, Spain

## Abstract

A specific family of proteins that participate in epigenetic regulation is the bromodomain (BRD) family of proteins. In this work, we aimed to explore the expression of the BRD family at a transcriptomic level in breast cancer, and its association with patient survival. mRNA level data from normal breast and tumor tissues were extracted from public datasets. Gene set enrichment analysis (GSEA) was performed to identify relevant biological functions. The KM Plotter Online tool was used to evaluate the relationship between the presence of different genes and patient clinical outcome. mRNA level data from HER2+ breast cancer patients sensible and resistant to trastuzumab were also evaluated. The BRD family was an enriched function. In HER2 positive tumors the combined analyses of BRD2, BAZ1A, TRIM33 and ZMYND8 showed a detrimental relapse free survival (RFS). Similarly, the combined analysis of BRD2, BAZ1A, PHIP, TRIM33, KMT2A, ASH1L, PBRM1, correlated with an extremely poor overall survival (OS). The prognosis was confirmed using an independent dataset from TCGA. Finally, no relation between expression of BRD genes and response to trastuzumab was observed in the HER2 population. Upregulation of some BRD genes is associated with detrimental outcome in HER2 positive tumors, regardless trastuzumab treatment.

## Introduction

Identification of oncogenic vulnerabilities with potential to be targeted therapeutically is a main goal in breast cancer^1,2^. Transcription factors (TFs) have been described as deregulated in cancer and linked with the oncogenesis of several malignancies including hematologic and solid tumors^3^. However, until very recently, pharmacological inhibition of their expression was not achievable.

Epigenetic regulation can indirectly modify the expression of TFs and, therefore, affect tumor progression^3^. A specific family of proteins that participate in the epigenetic modulation of transcription is the bromodomain (BRD) family of proteins^4^. Around 61 BRD modules can be combined in at least 42 different proteins^4^. Through the recognition of acetylated lysine residues on histone tails, they play a key role in the epigenetic control of gene transcription recruiting relevant transcriptional proteins and also acting as scaffolds for these proteins^4,5^. A subgroup that has gained attention is the BET family of proteins that compromised four members including BRD2, BRD3, BRD4 and BRDT (Bromodomain and Extraterminal proteins)^6,7^. Agents aiming to inhibit their function have been developed just recently, and they are currently in clinical evaluation at different stages, demonstrating a good toxicity profile^6,8^. In addition, they have shown activity in different solid tumors through the reduction of the expression of key TFs, including c-MYC or FOXM1, among others^9–11^.

Breast cancer is a heterogeneous disease, in which amplification of the tyrosine kinase receptor HER2, the expression of the estrogen receptor, or the lack of both molecular alterations guides their prognosis and therapeutic approach^2,12^. The HER2 positive subgroup represents around 20% of all tumors. Although novel anti-HER2 therapies, like the antibody pertuzumab or the antibody drug conjugate, trastuzumab-emtansine (TDM-1), have demonstrated to improve patient survival, most metastatic tumors are still incurable^13^. In a disease with multiple molecular alterations like breast cancer, where combinations of agents can augment clinical efficacy, the identification of novel vulnerabilities with potential to be exploited therapeutically is a main objective^14,15^.

In this work, we aimed to explore the expression of the BRD family of proteins in breast cancer and its association with patient survival. Using transcriptomic analyses, we observed that the BRD family of proteins was highly expressed in this tumor type, and the expression of some of them was associated with poor progression free and overall survival. Indeed, the combined expression of some of BRD genes strongly predicted poor outcome. Using an in silico evaluation we observed that the expression of these genes did not predict response to trastuzumab in patients. Finally, in other tumor subtypes, some of the family members were amplified and linked with prognosis.

## Results

### Functional transcriptomic profile of breast tumors identify BRD family as enriched

**Figure 1:**
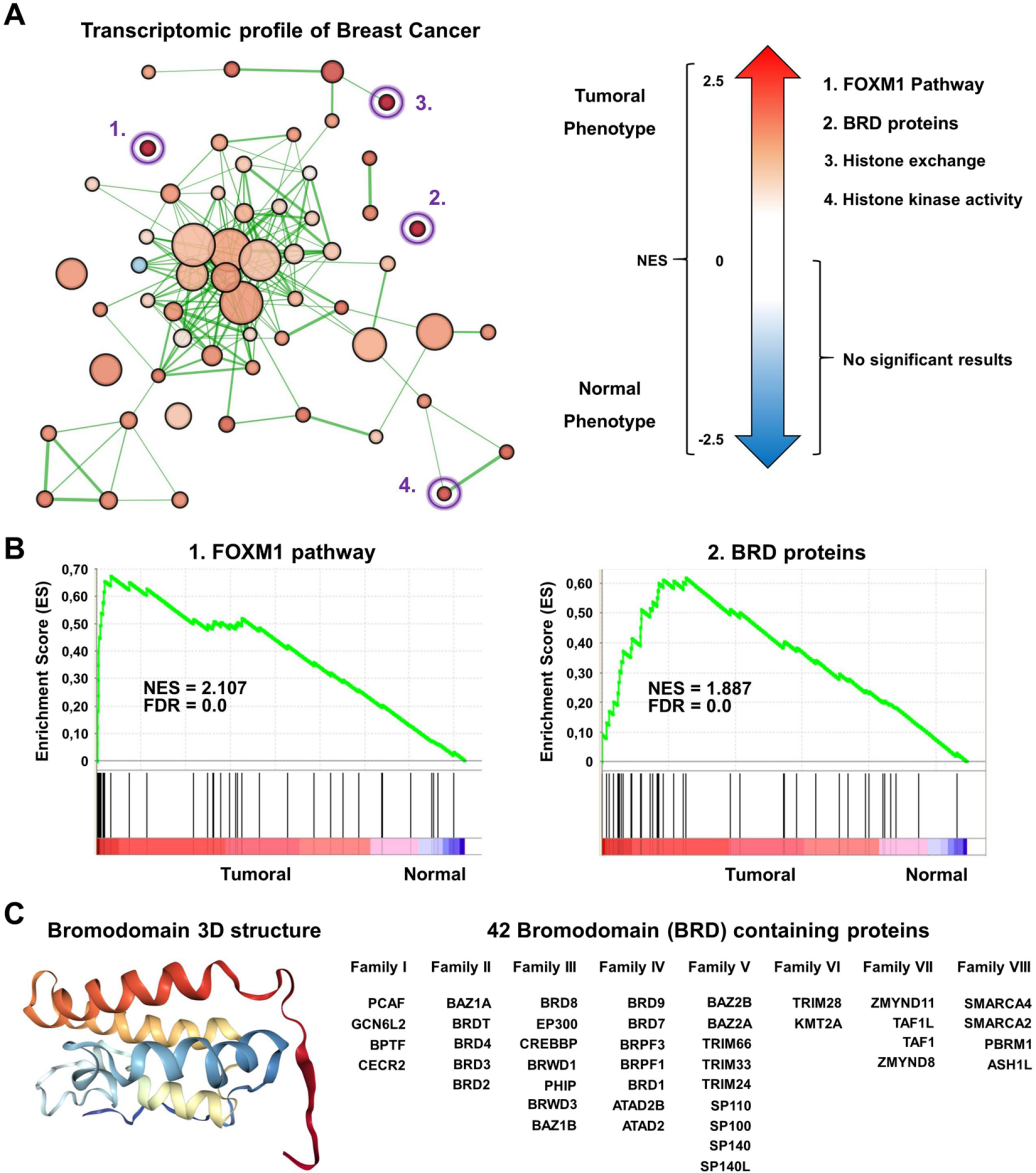
(**A**) Gene set enrichment network designed using Cytoscape (as described in material and methods), comparing normal versus breast tumoral tissue (all breast cancer subtypes). GeneSets overexpressed in the tumoral phenotype are displayed in shades of orange-red; and those overexpressed in the normal phenotype are displayed in shades of blue. (**B**) ES Score profile and locations of “FOXM1 pathway” and “BRD proteins” members on the rank ordered list. Positive NES defines tumoral phenotype enrichment. (**C**) 3D structure of the common BRD domain shared by all the proteins coded by these genes, and subclassification of the BRD proteins into their families.

First, we performed a transcriptomic analysis using several datasets (GSE21422, GSE26910, GSE3744, GSE65194, GSE42568) comparing normal breast with the four different breast cancer subtypes, including basal-like, luminal A and B, and HER2 enriched. GSEA identified several functions as deregulated, as shown in Fig. 1A. The “FOXM1 pathway” was the function more upregulated, but components of this family have already been studied in breast cancer^16,17^, so it was not the focus of our attention (Fig. 1A). Instead, we focused on BRD family genes, which were also clearly upregulated, and there are several therapeutic strategies against them in clinical development^6^. The BRD family had a net enrichment score of 1.8 supporting the relevance of this family (Fig. 1B). Figure 1C show the classification of the eight subfamilies of BRD containing proteins with the 3D structure.

### Upregulation of individual genes and outcome in breast cancer subtypes

**Figure 2:**
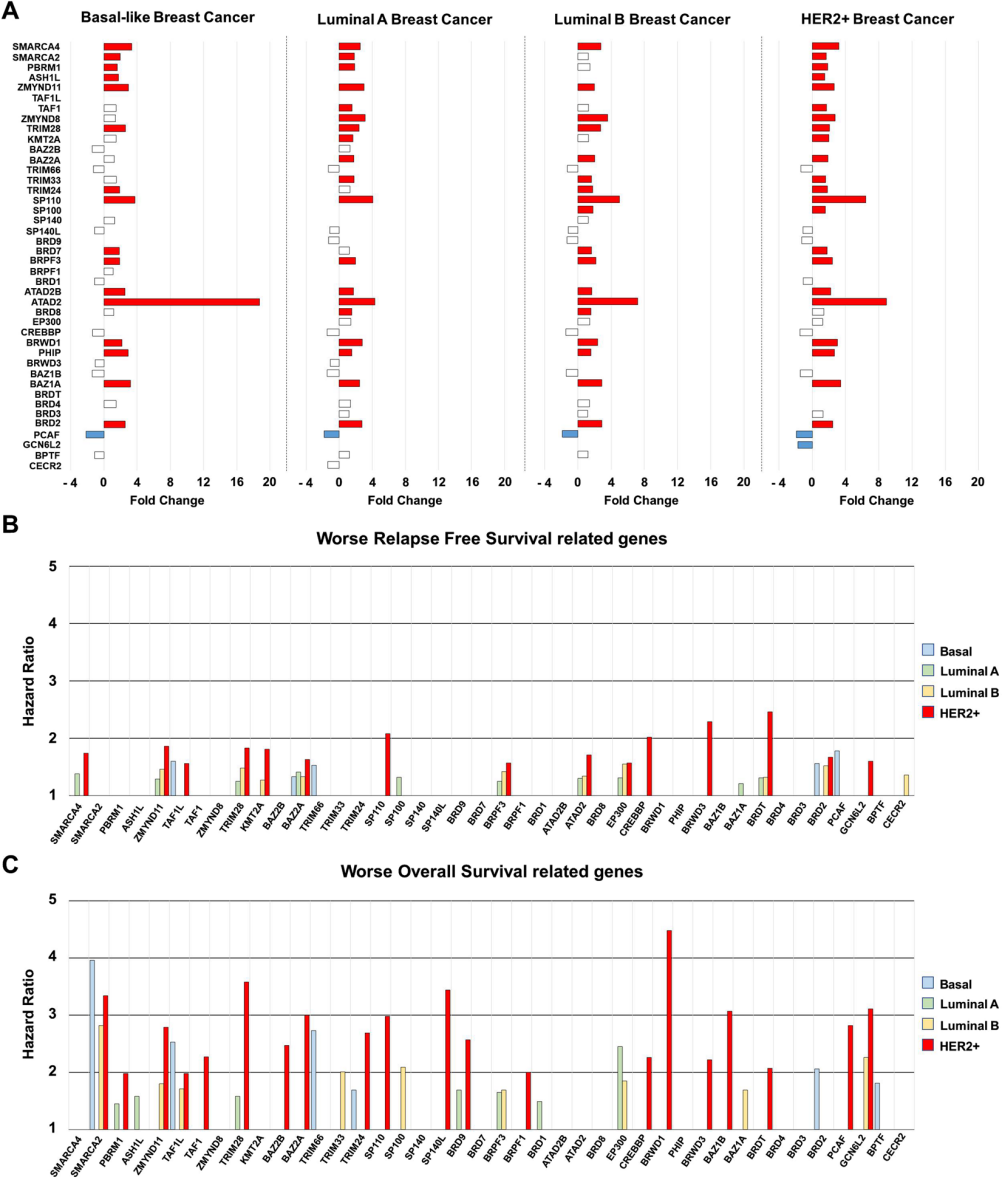
(**A**) Bar graph showing Fold Change expression values of genes included in the BRD family, comparing normal tissues and each breast cancer subtype. (**B**,**C**) Bar graph representing relapse free survival (RFS) hazard ratio values (**B**), and overall survival (OS) hazard ratio values (**C**), for each breast cancer subtype based on genes included in BRD family. Data was extracted from the KM plotter online tool as described in material and methods.

Analyses of raw transcriptomic data confirmed deregulation of most of the genes included in this family at different degree, depending on the breast cancer subtype (Fig. 2A).

Next, we intended to explore the role of these genes in relation to clinical outcome. For this purpose, we use the online tool KM Plotter, which associates gene expression level with prognosis^18^. Overexpression of several BRD genes was linked with detrimental relapse free survival (RFS) in breast tumors (Fig. 2B). This relation was also observed for overall survival (OS), being the HER2+ subgroup the subtype in which more BRD genes were related with patient’s outcome (Fig. 2C).

### Selection of genes associated with relapse free survival

**Figure 3:**
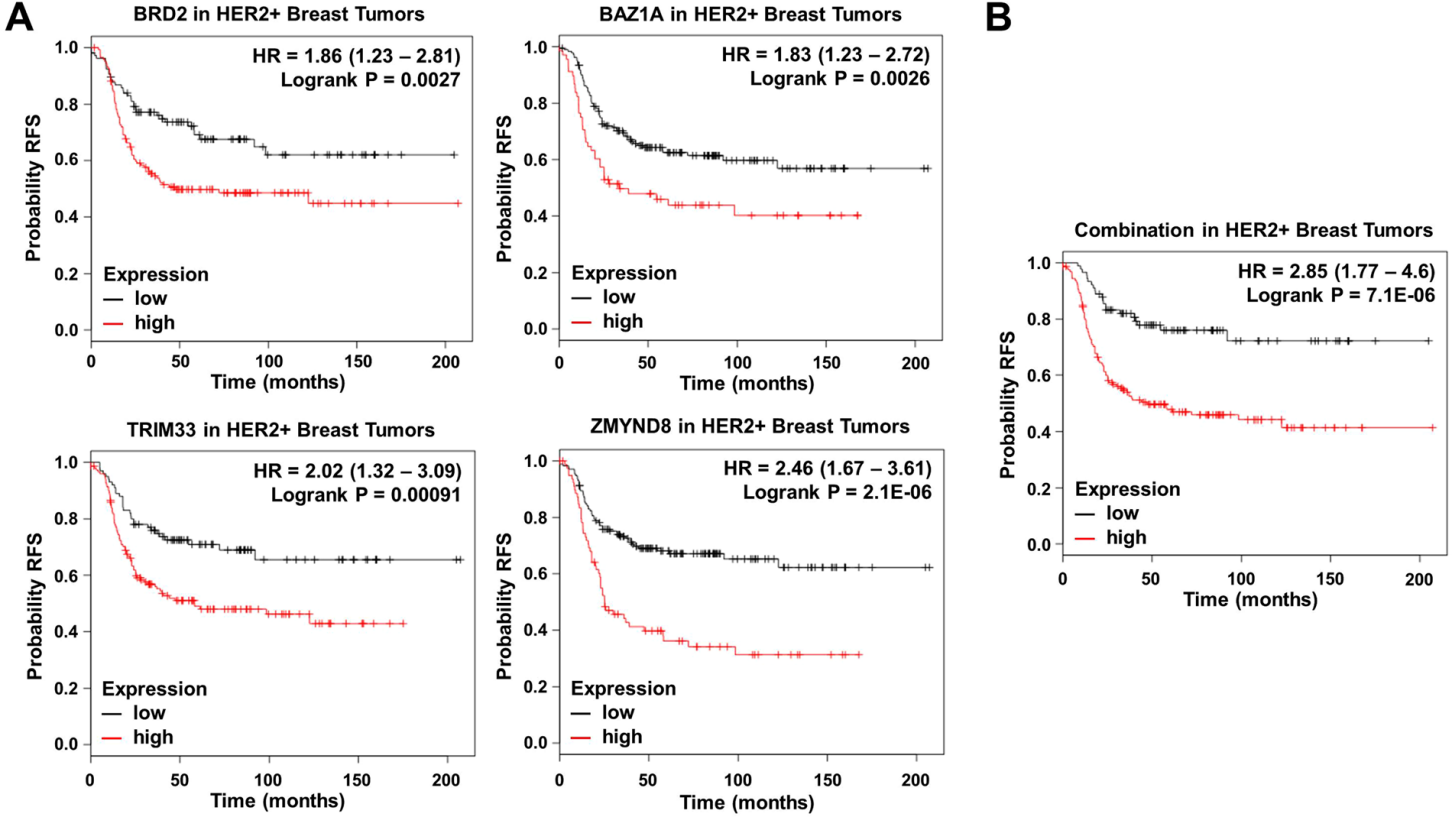
(**A**) Association of BRD2, BAZ1A, TRIM33 and ZMYND8 expression with relapse free survival in HER2+ tumors. (**B**) Association of the combined analyses of the four genes with overall survival in HER2+ tumors. Data was obtained as described in material and methods.

We selected those upregulated genes in HER2+ patients associated with worse RFS. Ten upregulated genes were linked with detrimental outcome: BRD2 [HR = 1.86 (1.23–2.81), log rank *p* = 0.0027], BAZ1A [HR = 1.83 (1,23–2,72), log rank *p* = 0.0026], PHIP [HR = 1.63 (1.1–2.42), log rank *p* = 0.014], BRD7 [HR = 1.57 (1.02–2.41), log rank *p* = 0.037], SP100 [HR = 1.51 (1.02–2.25), log rank *p* = 0.04], TRIM24 [HR = 1.57 (1.07–2.32), log rank *p* = 0.021], TRIM33 [HR = 2.02 (1.32–3.09), log rank *p* = 0.00091], ZMYND8 [HR = 2.46 (1.67–3.61), log rank *p* = 2.1e^−06^], ZMYND11 [HR = 1.67 (1.13–2.45), log rank *p* = 0.0089] and PBRM1 [HR = 1.6 (1.01–2.55), log rank *p* = 0.043] (Fig. 2B). Of note, the combined analysis of BRD2, BAZ1A, TRIM33, and ZMYND8, the genes more associated with poor outcome, showed a detrimental RFS that was higher than each gene analyzed individually [HR = 2.85 (1.77–4.6), log rank *p* = 7.1e^−06^] (Fig. 3A,B).

A multivariate analysis of this signature with variables like MKI67, ESR1 and ERB2 did not show any association with RFS except for ESR (p = 0.0199) (Supplementary Table 2A).

### A seven genes signature strongly predicted detrimental overall survival

**Figure 4:**
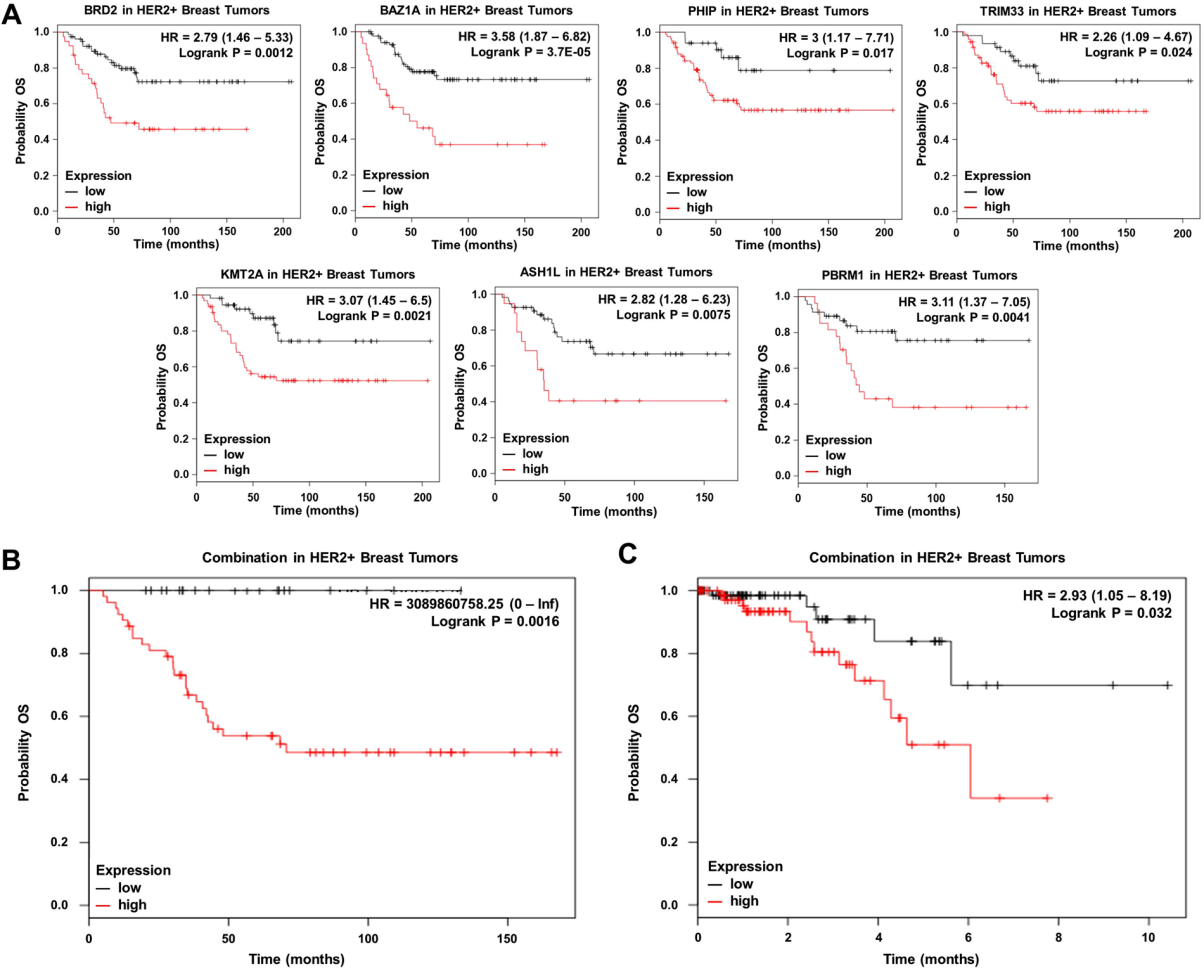
(**A**) Association of BRD2, BAZ1A, PHIP, TRIM33, KMT2A, ASH1L and PBRM1 individually with overall survival in HER2+ tumors. (**B**) Association of the combined analyses of the seven genes with overall survival in HER2+ tumors. (**C**) Association of the combined analyses of the seven genes with overall survival in HER2+ tumors using data from TCGA dataset.

Next, we selected those upregulated BRD genes in HER2+ patients related with worse OS. Eight genes met both criteria: BRD2 [HR = 2.79 (1.46–5.33), log rank *p* = 0.0012], BAZ1A [HR = 3.58 (1.87–6.82), log rank p = 3.7e^−05^], PHIP [HR = 3 (1.17–7.71), log rank *p* = 0.017], TRIM33 [HR = 2.26 (1.09–4.67), log rank *p* = 0.024], KMT2A [HR = 3.07 (1.45–6.5), log rank *p* = 0.0021], ZMYND8 [HR = 2.07 (1.08–3.97), log rank *p* = 0.025], ASH1L [HR = 2.82 (1.28–6.23), log rank *p* = 0.0075], and PBRM1 [HR = 3.11 (1.37–7.05), log rank *p* = 0.0041] (Fig. 2C and 4A). Finally, the combined analysis of BRD2, BAZ1A, PHIP, TRIM33, KMT2A, ASH1L, and PBRM1, the genes more associated with poor prognosis, showed an extremely detrimental survival [HR = 309860758.25 (0-inf), log rank *p* = 0.0016] (Fig. 4A,B). Notably, all patients with low expression of these genes were long term survivors. To confirm this result, we performed an independent validation using the TCGA breast cancer dataset^19^. Unfortunately, this dataset contains only 157 HER2 positive breast cancer patients with OS data. As seen in Fig. 4C, the seven genes signature demonstrated poor outcome regardless of this limited number of patients [HR = 2.93 (1.05–8.19), log rank *p* = 0.032.]

Finally we did a multivariate analysis with this seven gene signature including those variables included in the datasets from kmplotter such as MKI67, ESR1 and ERBB2. As shown in Supplementary Table 2B, not association with outcome was found, demonstrating the independence of the signature.

### Opportunities for target inhibition

Then, we searched for compounds in preclinical and clinical development that aim to inhibit the activity of the proteins coded by this family of genes. As shown in Supplementary Table 1, some of them are currently in preclinical or clinical development, like BI-9564 against BRD7, and OTX015 targeting BRD2, among others.

### BRD genes predict outcome in other breast cancer subtypes

Some BRD family members predicted worse outcome in other breast cancer subtypes, although to a lesser extent. In Basal-like patients, ZMYND11 was overexpressed and associated with worse both OS [HR = 3.77 (1.65–8.58), log rank *p* = 0.00069] and RFS [HR = 1.56 (1.15–2.12), log rank *p* = 0.0039] (Supplementary Fig. 1). In luminal A patients, overexpression of BAZ1A correlated with worse prognosis: OS [HR = 1.58 (1.09–2.29), log rank *p* = 0.015], RFS [HR = 1.25 (1.05–1.48), log rank *p* = 0.012] (Supplementary Fig. 2); and in luminal B tumors, we identified four genes linked with poor prognosis, BRD2: OS [HR = 1.8 (1.21–2.67), log rank *p* = 0.003], RFS [HR = 1.46 (1.19–1.77), log rank *p* = 0.00018]; ATAD2: OS [HR = 2.09 (1.03–4.23), log rank *p* = 0.038], RFS [HR = 1.37 (0.98–1.9), log rank *p* = 0.061]; BRD7: OS [HR = 1.69 (1.09–2.63), log rank *p* = 0.017], RFS [HR = 1.42 (1.17–1.74), log rank *p* = 0.00051] and TRIM24: OS [HR = 1.85 (1.26–2.72), log rank *p* = 0.0014], RFS [HR = 1.55 (1.26–1.9), log rank *p* = 2.6e^−05^] (Supplementary Fig. 3). Some of these genes were amplified in breast tumors, like ZMYND11, which was found to be amplified in 28% of basal-like tumors (Table 1).

**Table 1:** Percentage of clinical cases with molecular alterations described in the genes associated with clinical outcome classified by breast cancer subtypes. Data was obtained as described in material and methods.

		Gene	BRD2	BAZ1A	PHIP	TRIM33	ZMYND8	PBRM1	ZMYND11	ATAD2	BRD7	TRIM24
Breast Cancer (METABRIC, 2051 samples)	No classification	Amplification	0.98	1.41	1.07	0.73	4.29	0.1	4.05	24.23	1.12	2.15
		Mutation	—	—	—	—	—	1.66	—	—	—	—
		Deep Deletion	0.05	0.54	0.05	—	—	0.15	0.39	—	0.1	—
		Multiples alterations	—	—	—	—	—	—	—	—	—	—
Breast Invasive Carcinoma (TCGA, Cell 2015) (818 samples)	Her2—positive breast tumors (120 samples)	Amplification	0.83	2.5	—	—	4.17	—	2.5	26.67	—	—
		Mutation	—	1.67	0.83	0.83	0.83	2.5	—	0.83	0.83	1.67
		Deep Deletion	—	—	—	—	—	0.83	0.83	—	0.83	0.83
		Multiples alterations	—	—	—	—	—	—	—	—	—	—
	Invasive ductal cancer (Luminal A) (201 samples)	Amplification	1	1.99	0.5	—	3.98	0.5	0.5	11.44	1	—
		Mutation	—	—	—	0.5	1	0.5	0.5	1	0.5	1
		Deep Deletion	—	—	0.5	—	—	0.5	—	—	—	—
		Multiples alterations	—	—	—	—	—	—	—	—	—	—
	Invasive ductal cancer (PAM50 Luminal B) (122 samples)	Amplification	—	5.74	0.82	0.82	5.74	—	—	28.69	0.82	0.82
		Mutation	0.82	2.46	0.82	0.82	—	0.82	—	1.64	0.82	0.82
		Deep Deletion	—	—	—	—	—	0.82	—	—	0.82	—
		Multiples alterations	—	—	—	—	—	—	—	1.64	—	—
	Invasive ductal cancer (PAM50 Basal—like) (107 samples)	Amplification	4.67	2.8	—	0.93	5.61	—	28.04	38.32	—	4.67
		Mutation	0.93	2.8	0.93	—	—	0.93	—	0.93	—	0.93
		Deep Deletion	—	—	—	0.93	—	—	—	—	2.8	0.93
		Multiples alterations	—	—	—	—	—	—	—	—	—	—

### BRD family is not associated with resistance to trastuzumab

**Figure 5:**
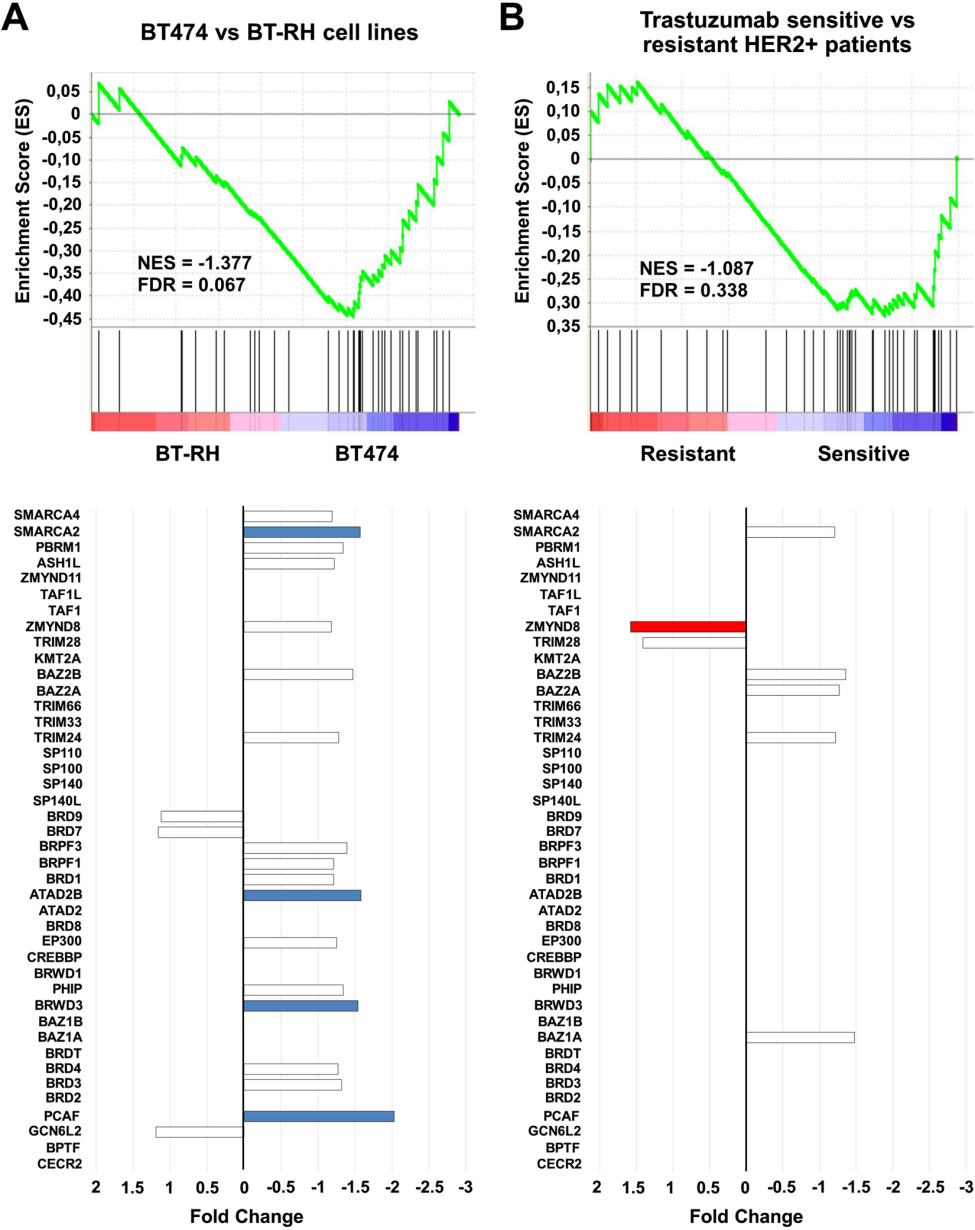
(**A**) Enrichment Score (ES) comparing BT474 and BT-RH cell lines (upper panel). Bar graph showing Fold Change expression values of genes included in the BRD family, comparing BT474 and BT-RH (lower panel). (**B**) ES Score comparing long trastuzumab responder patients and non-responders patients (upper panel). Bar graph showing Fold Change expression values of genes included in the BRD family, comparing long trastuzumab responder patients and non-responders patients (lower panel).

Finally, we explored if the relationship with prognosis was due to an implication of these proteins in resistance to trastuzumab therapies, or if they were just a molecular alteration present in HER2 breast tumors. For this purpose, we used a trastuzumab resistant cell line (BT-RH), generated in our laboratory as described in Material and Methods. First, we evaluated the transcriptional status of BRD genes in this cell line compared to parental non-resistant BT474 cells. Figure 5A shows that BRD family was enriched in BT474 comparing to BT-RH cells. Indeed, individualized analysis of each gene also indicated that PCAF, BRWD3, ATAD2B, and SMARCA2 were more than 1.5-fold underexpressed in BT-RH.

Next, we evaluated if these results could be reproduced in patients. To do so, we used data from the Long-HER study contained at GSE44272, in which patients treated with trastuzumab were classified in two groups: responders and not responders^20^. No clear enrichment was observed in trastuzumab resistant patients (Fig. 5B). Only ZMYND8 was found to be overexpressed in patients with worse response to Trastuzumab. Finally, by using an integrated response database (as described in material and methods), we confirmed that the BRD family of proteins was not directly related to a worse response to Trastuzumab (Table 2).

**Table 2:** Predictive response to Trastuzumab treatment values of genes both overexpressed and related to worse outcome in HER2+ patients obtained as described in material and methods.

	Gene symbol	Gene Name	Mann-Whitney test p-value	Fold Change	AUC	P-value
Family II	**BRD2**	Bromodomain containing 2	0.063	**0.9**	0.579	**0.031**
	**BAZ1A**	Bromodomain adjacent to zinc finger domain protein 1A	0.072	**0.94**	0.577	**0.035**
Family III	**PHIP**	Pleckstrin homology domain interacting protein	0.18	**0.88**	0.557	0.092
Family IV	**BRD7**	Bromodomain containing 7	0.11	**0.86**	0.568	0.054
Family V	**SP100**	SP100 nuclear antigen	0.16	**0.83**	0.56	0.078
	**TRIM24**	Tripartite motif containing 24	0.32	**0.9**	0.542	0.16
	**TRIM33**	Tripartite motif containing 33	0.87	**1**	0.508	0.44
FamilyVI	**KMT2A**	Lysine (K)-specific methyltransferase 2A; uncharacterized LOC101929115	**0.023**	**1**	0.597	**9.90E-03**
Family VII	**ZMYND8**	Zinc finger, MYND-type containing 8	0.38	**1.1**	0.537	0.19
	**ZMYND11**	Zinc finger, MYND-type containing 11	**0.02**	**0.85**	0.599	**9.30E-03**
Family VIII	**ASH1L**	Ash1 (absent, small, or homeotic)-like (Drosophila)	0.25	**0.9**	0.562	0.12
	**PBRM1**	Polybromo 1	0.14	**1.2**	0.579	0.068

## Discussion

In this article, we identify a family of proteins that can predict detrimental survival in breast cancer, particularly in HER2 positive tumors, regardless the treatment of choice. Some of these genes were also implicated in patients’ outcome in other breast cancer subtypes.

Breast cancer is a heterogeneous disease in which several molecular alterations exist, and, among them, the overexpression of HER2 is observed in around 20% of breast tumors^13^. HER2 is an oncoprotein that is necessary to initiate the tumor, as it is expressed in premalignant lesions, but is not sufficient to promote cancer progression and dissemination, as it requires the presence of other molecular alterations^14^ In this context, the identification of additional vulnerabilities beyond the mere presence of HER2 is a main objective.

Anti-HER2 strategies are given during all period of time of the disease, contributing to the improvement in survival of patients with advanced disease^21^. However, administration of other treatments, like chemotherapy or anti-estrogens, in estrogen receptor positive tumors (luminal B tumors), can clearly augment the efficacy of trastuzumab alone^13^. This demonstrates, at a clinical level, that other oncogenic vulnerabilities beyond the presence of HER2 do exist, and the search of therapeutic strategies against them should be pursued. Examples of studies in clinical development using this approach are those that evaluate CDK4/6 inhibitors in combination with anti-HER2 therapies^22^. Of note, this potential effect does not involve resistance to the current therapy.

In our article, we describe a family of genes linked with detrimental prognosis in HER2 positive tumors, but not associated with response to trastuzumab, what suggests and additional vulnerability in HER2 cancers. Of note, we observed the upregulation of some of these genes associated with outcome in some other subtypes. Of note multivariate analyses including MKI67, ESR1 and ERBB2 measured by immunohistochemistry did not show any association with clinical outcome, except for ESR1 with RFS. This last finding suggests that hormone receptor positive tumors have a slightly different behavior, probably as they belong to the Luminal B subtype.

Some of them can be inhibited pharmacologically, like is the case of BRD2 or BRD7, and it is anticipated that specific drugs against many others will be developed in the near future. BRD2 is a key TF with a relevant role in cell cycle progression; indeed, complete knockout of BRD2 in mice induces lethality^23^. Other identified gene was ATAD2, which has been previously implicated in the transcription regulation of genes by the ER^24,25^; although no data exist in HER2 positive breast tumors.

In line with this, we have identified that ZMYND11 is amplified in 28% of basal-like tumors and associated with detrimental prognosis, suggesting that manipulations of this gene could be therapeutically exploited. However, data regarding the role of this protein in breast cancer support that low levels are associated with detrimental prognosis, demonstrating its different function and role depending on the tissue and tumor of origin^26^.

This work is the result of an extensive in silico study, but we acknowledge some limitations, as not preclinical work with cell lines or animal models has been performed. In addition no confirmation using immunohistochemistry has been done. Then, further assessment of the effect on the inhibition of each of the genes identified is required for future studies.

In conclusion, here we have mapped the expression of the BRD family of proteins in breast cancer, particularly in HER2 positive tumors, and showed their association with outcome. The data presented here opens the opportunity for further studies exploring the functional effect of the inhibition of these genes on oncogenic properties in different breast cancer subtypes.

## Methods

### Whole genome transcription profiling and Gene-set enrichment analyses

mRNA data from normal breast and tumor tissue (basal-like, Luminal A, Luminal B and HER2+) were extracted from public datasets (GEO DataSet accession numbers: GSE21422, GSE26910, GSE3744, GSE65194 and GSE42568). Affymetrix CEL files were downloaded and analyzed with the Affymetrix Expression Console. We further performed gene set enrichment analysis (GSEA) to identify transcription related functions that varied between normal and tumor tissues. 58 Gene sets were collected from the Molecular Signatures Database (MSigDB) (http://www.broadinstitute.org/gsea/msigdb/), including 53 from Gene Ontology (GO) biological process and one constructed by ourselves with BRD family proteins; data were analyzed by GSEA with parameter set to 1.000 gene-set permutations. This analysis yields an enrichment score. When this score is positive (e.g. the gene set is overrepresented by top ranked genes) the gene set is considered upregulated. Contrary, the gene set is considered downregulated when the score is negative. The network of gene sets interactions was constructed by using Cytoscape software (version 3.4.0). Affymetrix CHP files were analyzed with Affymetrix Transcriptome Analysis Console 3. Only genes with maximum 0.05 p-value differential expression between control and tumor were selected.

### Outcome analyses

The KM Plotter Online Tool (http://www.kmplot.com)^18^ was used to evaluate the relationship between the presence of different genes and patient clinical outcomes in different breast cancer subtypes. This publicly available database allowed us to investigate overall survival (OS) and relapse-free survival (RFS) in basal-like, luminal A, luminal B, HER2+ and triple negative breast cancers. Breast cancer subtypes were defined depending of their expression of ESR1 and HER2. Thus, Basal-like were defined as ESR1−/HER2−; Luminal A as ESR1+/HER2−/MKI67 low; Luminal B as ESR1+/HER2−/MKI67 high or ESR1+/HER2+; HER2+ as ESR1−/HER2+; and Triple negative as ER−/PR−/HER2−. The best performing threshold between low and high expression was used as a cutoff.

TCGA dataset^19^ were used to confirm of RFS and OS of the identified signatures.

### Transcriptomic profiling of trastuzumab resistant cell lines and patients

An *in vitro* cellular model of trastuzumab resistance was generated from BT474 cells, grown in Dulbecco´s modified Eagle medium (DMEM) containing antibiotics and 10% FBS. To obtain such model, BT474 cells, were plated at low density and grown in the presence of 50 nM trastuzumab for 6 months. Trastuzumab-resistant cells were pooled, generating a cell population that was named BT-RH (for “Resistant to Herceptin™”) cells. Total RNA was extracted and purified using the RNeasy Mini Kit (Qiagen). Biotinylated complementary RNA was then synthesized (Enzo Life Sciences) and hybridized to HG-U133 plus 2.0 GeneChip oligonucleotide arrays (Affymetrix), according to the manufacturer’s instructions. Quantitation of fluorescence intensities of probesets was done using the GenArray Scanner (Hewlett Packard). This process was repeated three independent times. For the microarray data analysis, Affymetrix CEL files generated in each different experiment were imported into the Expression Console software and normalized using the RMA algorithm.

mRNA data from HER2+ breast cancer tissues from patients sensible and resistant to treatment with Trastuzumab were extracted from a public dataset (GEO DataSet accession numbers: GSE44272). Affymetrix CEL files were downloaded and analyzed with Affymetrix Expression Console. Affymetrix CHP files from both cell lines and patients tissues were analyzed with Affymetrix Transcriptome Analysis Console 4. Only genes with maximum 0.05 p-value differential expression between control and tumor were selected.

### Prediction of Trastuzumab response analyses

We used a database of transcriptomic datasets with available response and treatment data used to evaluate the relationship between the expression of those genes of interest and patients response to different treatments, including Trastuzumab. A Pubmed search using the keywords “breast cancer”, “survival”, “treatment”, and “response”, lead us to the identification of 2,108 breast cancer samples who received chemotherapy treatment and for whom the gene expression was measured using Affymetrix HGU133A and HGU133A plus 2.0 microarrays. We used those in which trastuzumab was included in the treatment.

### Statistical analyses

Kaplan-Meier plots were drawn to visualize the survival differences. Cox proportional hazards regression was computed to explore the association between gene expression and outcomes. Multiple genes were combined into a signature by using their mean expression. Statistical significance was defined as p < 0.05. In Gene Set Enrichment Analysis (GSEA), FDR < 0.25 was considered a statistically significant difference. Multivariate analysis was performed including MKKI67, ESR1 and ERBB2 using the dataset from kmplotter and measured by immunohistochemistry staining.

## Supplementary information

Supplementary Information

## Data Availability

The datasets analyzed during the current study are available in GSE21422 (https://www.ncbi.nlm.nih.gov/geo/query/acc.cgi?acc=GSE21422), GSE26910 (https://www.ncbi.nlm.nih.gov/geo/query/acc.cgi?acc=GSE26910), GSE3744 (https://www.ncbi.nlm.nih.gov/geo/query/acc.cgi?acc=GSE3744), GSE65194 (https://www.ncbi.nlm.nih.gov/geo/query/acc.cgi?acc=GSE65194), GSE42568 (https://www.ncbi.nlm.nih.gov/geo/query/acc.cgi?acc=GSE42568), and GSE44272 (https://www.ncbi.nlm.nih.gov/geo/query/acc.cgi?acc=GSE44272) repositories. The normalization of the CEL files of these 5 studies resulted in our breast cancer functional transcriptome profile generated during the current study. Microarray data from BT474 and BTRH are now available through the GEO repository database in GSE119397.
