# Supplementary material for: Mapping Bromodomains in breast cancer and association with clinical outcome
Source: Sci Rep. 2019 Apr 5;9:5734. doi: 10.1038/s41598-019-41934-3 (PMC6450889; doi:10.1038/s41598-019-41934-3)
Supplement: Supplementary file 1 — Supplementary Information [file 41598_2019_41934_MOESM1_ESM.docx]

**Mapping Bromodomains in breast cancer and association with clinical outcome**

**Javier Pérez-Pena^1^, Raquel Páez^1^, Cristina Nieto-Jiménez^1^, Verónica Corrales Sánchez^1^, Eva M. Galan-Moya^1^, Atanasio Pandiella^2^, Balázs Győrffy^3^ and Alberto Ocana^1^***

*1 Translational Research Unit and Translational Oncology Laboratory, Albacete University Hospital and Centro Regional de Investigaciones Biomedicas, Castilla‐La Mancha University (CRIB‐UCLM), Albacete, Spain.*

*2 Instituto de Biología Molecular y Celular del Cáncer and CIBERONC. CSIC-Universidad de Salamanca, Salamanca, Spain.*

*3 Semmelweis University 2nd Department of Pediatrics, Budapest, Hungary and MTA TTK Lendület Cancer Biomarker Research Group, Institute of Enzymology, Budapest, Hungary.*

|  | **Gene symbol** | **Gene name** | **Gene overexpression and worse outcome by tumor subtype** | **Compound** |
| --- | --- | --- | --- | --- |
| **Family II** | BRD2 | Bromodomain containing 2 | Luminal A/Luminal B/HER2+ | *OTX015* |
|  |  |  |  | *Mivebresib (ABBV-075)* |
|  |  |  |  | *PLX51107* |
|  |  |  |  | *I-BET-762 (GSK525762)* |
|  |  |  |  | (+) JQ1 |
|  |  |  |  | PFI-1 (PF-6405761) |
|  |  |  |  | Bromosporine |
|  |  |  |  | I-BET151 (GSK1210151A) |
|  |  |  |  | I-BET726 (GSK1324726A) |
|  |  |  |  | ARV-771 |
|  |  |  |  | MS436 |
|  |  |  |  | PROTAC 11 |
|  | BAZ1A | Bromodomain adjacent to zinc finger domain protein 1A | Luminal A/Luminal B/HER2+ | - |
| **Family III** | PHIP | Pleckstrin homology domain interacting protein | Basal/Luminal A/Luminal B/HER2+ | - |
|  | BRWD1 | Bromodomain and WD repeat domain containing 1 | Basal | - |
| **Family IV** | ATAD2 | ATPase family, AAA domain containing 2 | Luminal A/Luminal B | GSK8814 |
|  |  |  |  | BAY-850 |
|  | BRD7 | Bromodomain containing 7 | Luminal B/HER2+ | BI-9564 |
|  |  |  |  | BI-7273 |
|  |  |  |  | TP-472 |
|  |  |  |  | LP99 |
| **Family V** | SP100 | SP100 nuclear antigen | Luminal B/HER2+ | - |
|  | SP110 | SP110 nuclear body protein | HER2+ | - |
|  | TRIM24 | Tripartite motif containing 24 | Luminal B | IACS-9571 |
|  | TRIM33 | Tripartite motif containing 33 | HER2+ | - |
| **Family VI** | KMT2A | Lysine (K)- specific methyltransferase 2A | HER2+ | - |
|  | TRIM28 | Tripartite motif containing 28 | Luminal A/Luminal B | - |
| **Family VII** | ZMYND8 | Zinc finger, MYND-type containing 8 | Luminal A/Luminal B/HER2+ | - |
|  | ZMYND11 | Zinc finger, MYND-type containing 11 | Basal/Luminal B/HER2+ | - |
| **Family VIII** | ASH1L | Ash1 (absent, small or homeotic)-like (Drosophila) | Basal/HER2+ | - |
|  | PBRM1 | Polybromo 1 | HER2+ | PFI-3 |
|  | SMARCA2 | SWI/SNF related, matrix associated, actin dependent regulator of chromatin, subfamily a, member 2 | Basal | PFI-3 |
|  | SMARCA4 | SWI/SNF related, matrix associated, actin dependent regulator of chromatin, subfamily a, member 4 | Luminal B | PFI-3 |

Supplementary Table 1

|  |  | **p-value** | **Hazard Ratio** |
| --- | --- | --- | --- |
| **RFS** | MKI67 | 0.5063 | 0.86 (0.55 - 1.34) |
|  | ESR1 | **0.0199** | 0.59 (0.38 - 0.92) |
|  | ERBB2 | 0.8437 | 1.05 (0.63 - 1.76) |

**A**

**B**

|  |  | **p-value** | **Hazard Ratio** |
| --- | --- | --- | --- |
| **OS** | MKI67 | 0.4412 | 1.93 (0.36 - 10.36) |
|  | ESR1 | 0.0686 | 0.21 (0.04 - 1.13) |
|  | ERBB2 | 0.1632 | 4.24 (0.56 - 32.33) |

Supplementary Table 2

*
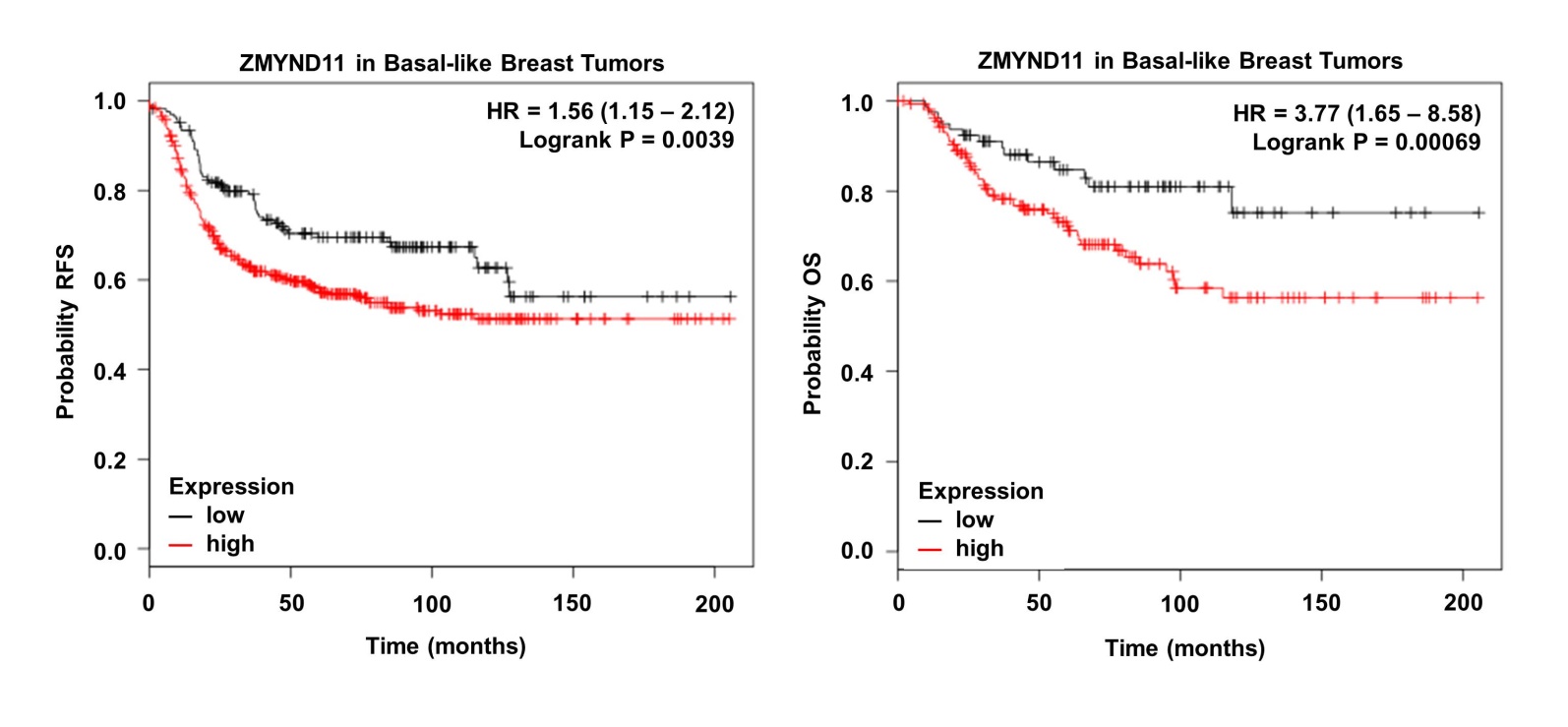
*

Supplementary Figure 1


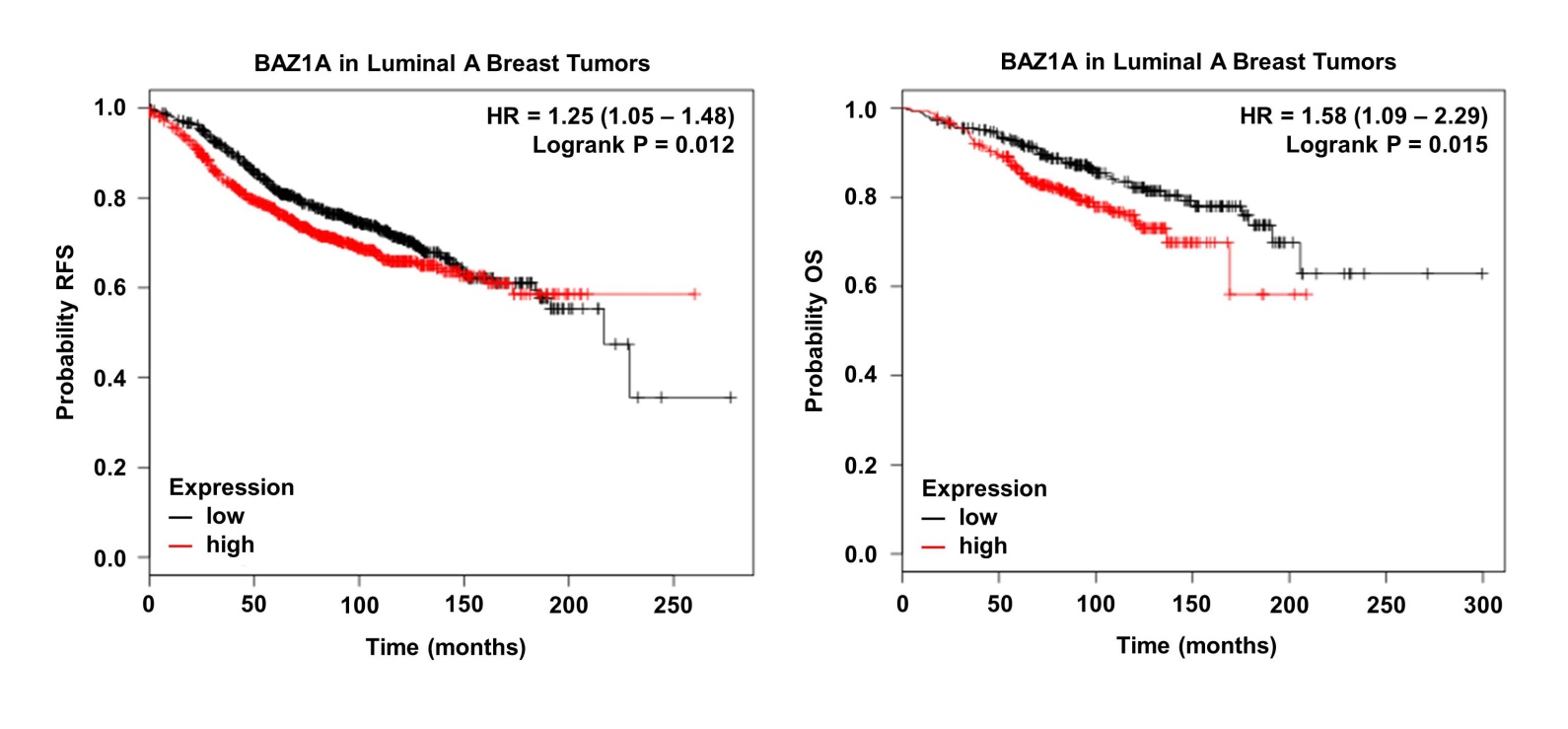


Supplementary Figure 2


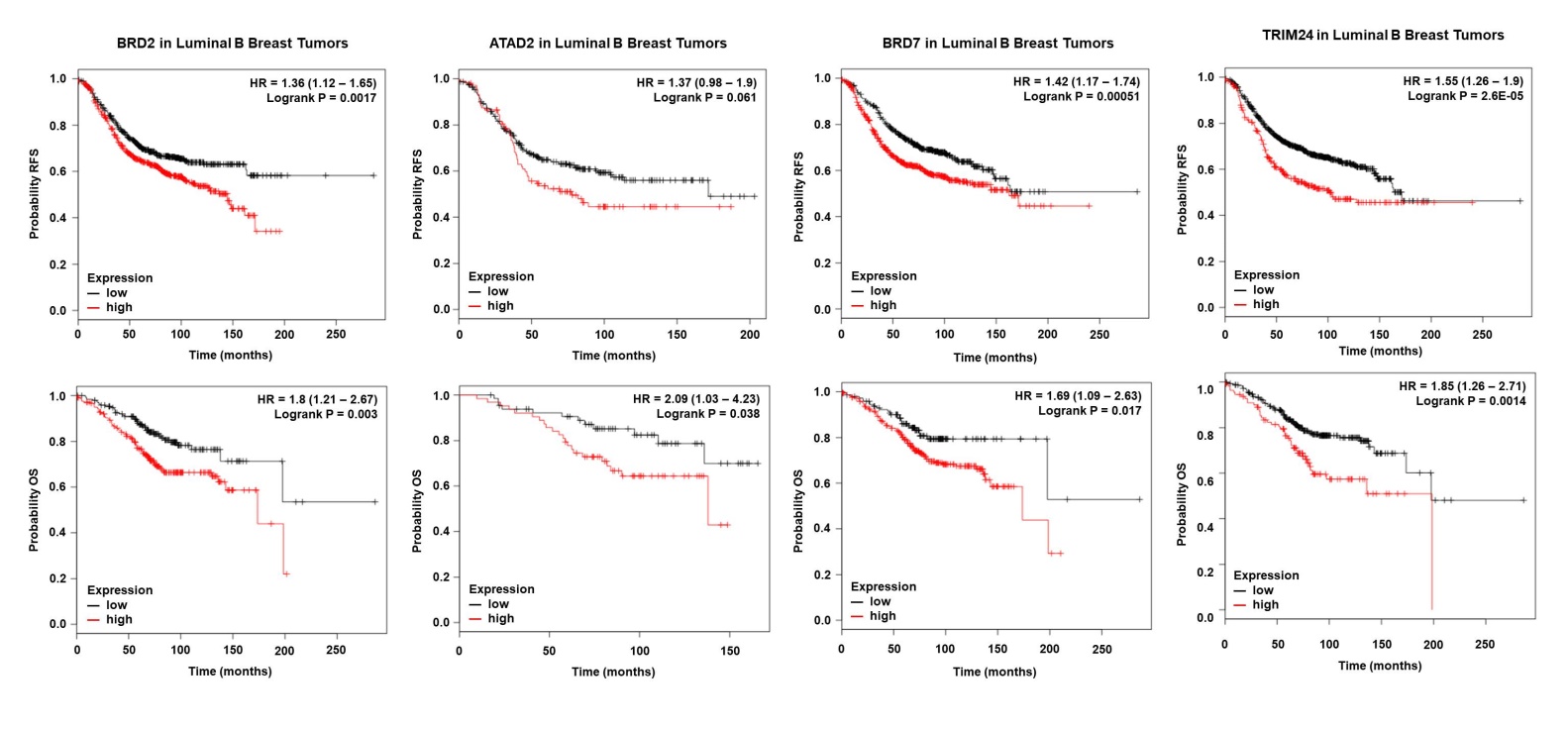


Supplementary Figure 3

Supplementary Table 1. Compounds in preclinical and clinical development that inhibit the activity of proteins coded by genes overexpressed, and related to worse outcome.

Supplementary Table 2. (A) Relapse free survival multivariate analysis for MKI67, ESR1 and ERBB2 facing BRD2, BAZ1A, TRIM33, and ZMYND8 combined expression in HER2+ tumors. (B) Overall survival multivariate analysis for MKI67, ESR1 and ERBB2 facing BRD2, BAZ1A, PHIP, TRIM33, KMT2A, ASH1L and PBRM1 combined expression in HER2+ tumors.

Supplementary Figure 1. Association of ZMYND11 with relapse free survival (left panel) and overall survival (right panel) in basal-like tumors.

Supplementary Figure 2. Association of BAZ1A individually with relapse free survival (left panel) and overall survival (right panel) in luminal A tumors.

Supplementary Figure 3. Association of BRD2, ATAD2, BRD7 and TRIM24 individually with relapse free survival (upper panel) and overall survival (lower panel) in luminal B tumors.
